# Thermal Conductivity in Concrete Samples with Natural and Synthetic Fibers

**DOI:** 10.3390/ma17040817

**Published:** 2024-02-08

**Authors:** Lucas Daza-Badilla, René Gómez, Ramón Díaz-Noriega, Siva Avudaiappan, Krzysztof Skrzypkowski, Erick I. Saavedra-Flores, Waldemar Korzeniowski

**Affiliations:** 1 Faculty of Engineering, Universidad de Concepción, Concepcion 4030000, Chile; ldaza2016@udec.cl (L.D.-B.); rdiaz@udec.cl (R.D.-N.);; 2 Faculty of Construction Sciences, Universidad Tecnológica Metropolitana, Santiago 7501370, Chile; 3 Faculty of Civil Engineering and Resource Management, AGH University of Krakow, 30-059 Krakow, Poland; walkor@agh.edu.pl; 4 Departamento de Ingeniería en Obras Civiles, Universidad de Santiago de Chile, Santiago 4070371, Chile

**Keywords:** concrete, natural fiber, thermal conductivity, transient line source

## Abstract

One crucial property of concrete, particularly in construction, is its thermal conductivity, which impacts heat transfer through conduction. For example, reducing the thermal conductivity of concrete can lead to energy savings in buildings. Various techniques exist for measuring the thermal conductivity of materials, but there is limited discussion in the literature about suitable methods for concrete. In this study, the transient line source method is employed to evaluate the thermal conductivity of concrete samples with natural and synthetic fibers after 7 and 28 days of curing. The results indicate that concrete with hemp fiber generally exhibits higher thermal conductivity values, increasing by 48% after 28 days of curing, while synthetic fibers have a minimal effect. In conclusion, this research opens the door to using natural alternatives like hemp fiber to improve concrete’s thermal properties, providing alternatives for thermo-active foundations and geothermal energy piles which require high thermal conductivities.

## 1. Introduction

Concrete is a widely used material worldwide, with an annual production of over ten billion tons [1]. Its various applications include usage in buildings, bridges, tunnels, industrial pavements, and many other types of structures. An important property of concrete in some applications is thermal conductivity. This is the primary property that affects heat transfer based on conduction in concrete. At typical operating temperatures, heat primarily moves through concrete material through conduction [2]. Depending on the use of the concrete, it is sought that it has a greater or lesser thermal conductivity. The use of concrete as a construction material in buildings requires lower thermal conductivities because it improves the thermal insulation characteristics of buildings and allows for a lower energy consumption [3]. On the other hand, the concrete used for thermo-active foundations and geothermal energy piles requires high thermal conductivities for better heat transfer between the ground and the heat exchange circuit of the foundation [4,5]. 

In concrete, thermal conductivity can be modified by incorporating different types of aggregates [6,7]. The volume of aggregate in concrete generally accounts for approximately 60–80% of its overall composition. In particular, increasing the volume fraction of coarse aggregate while maintaining a constant sand ratio can elevate the k-value of concrete [8]. The thermal conductivity of rocks used as aggregate in concrete ranges from 1.163 to 8.6 W/m°K [9]. On the other hand, there is a notable connection between the unit weight of concrete and its thermal conductivity value [10]. When replacing conventional aggregate with lightweight foamed concrete, the thermal conductivity of the resulting concrete can decrease due to the porosity of lightweight aggregates. For example, a 1% increase in concrete porosity can cause a 0.6% decrease in thermal conductivity [11].

In addition, studies have evaluated which variables affect thermal conductivity in concrete. In one of these studies, seven variables affecting the thermal conductivity of cement paste, mortar, and concrete were examined [6]. These seven variables included the moisture level of the sample, age, temperature, water–cement (*w*/*c*) ratio, proportion of fine aggregate, type of admixture, and overall aggregate volume fraction. It was found that moisture content, specimen condition, and aggregate volume fraction were the key determinants of concrete’s thermal conductivity. In contrast, the *w*/*c* ratio and admixture type were the primary influencing factors of the k-value of cement paste and mortar. Additionally, voids within the concrete have a notable impact on both the mechanical and thermal properties of the material [7]. In [12], the thermal conductivity reduced by 49.1% for the mix containing 40% of waste polypropylene. Moreover, the thermal conductivity decreased by 11% and 47% using 50% of plastic waste [13].

Regarding moisture, research has been conducted [14] based on experimental measurements and self-consistent scheme modeling. The thermal conductivity of the materials under examination varies between 90 and 160 mW/(m°K) at 23 °C with 50% of relative humidity. The influence of density on thermal conductivity is significantly more pronounced than the influence of moisture content. It is demonstrated that as density increases by two-thirds, thermal conductivity increases by approximately 54%, whereas it only increases by less than 15–20% when transitioning from a dry state to 90% relative humidity. Similarly, there are certain studies that approach the thermal conductivity of concrete from a different perspective, such as in the case of [15], where graphene-based materials were chosen to enhance the thermal conductivity of cement and address the thermal expansion and cracking behavior of concrete. Furthermore, it was demonstrated that graphene oxide with a high oxygen concentration can improve both the mechanical and thermal properties of cement-based materials.

Additionally, fibers are also frequently used in concrete. In particular, synthetic fibers possess high electrical and thermal conductivity, along with a relatively low coefficient of thermal expansion [16,17,18,19]. This inherent property of synthetic fibers makes them well suited for applications in the aerospace, electronics, and automotive sectors. In [20], the authors found that the addition of polypropylene synthetic fiber did not have a significant effect on the thermal conductivity of lightweight concrete. Also, it was found that in lightweight foamed concrete [21], the addition of polypropylene fibers decreases the thermal conductivity of the material. In particular, the greater the number of polypropylene fibers added to the material, the greater the decrease in thermal conductivity. 

Concrete with hemp fiber, a bio-aggregate building material used in walls, floors, and roofs [22], is environmentally friendly and porous. The thermal conductivity of hemp is more significantly affected by density than by water content. It was found that as the hemp content in the concrete increases, the thermal conductivity decreases. It was also found that for sprayed hemp concrete walls, the thermal conductivity varies from 0.116 to 0.145 W/m°K when the densities range from 374 to 450 kg/m^3^. Therefore, hemp can be a valuable material in the manufacturing of sustainable and durable construction materials with excellent thermal properties depending on its characteristics. Some other studies indicate that hemp has a positive effect on the thermal conductivity of hemp concretes [17,21]. 

In general, there are various studies regarding hempcrete, ranging from hygrothermal to acoustic qualities. Some research on these topics includes that of Piot et al. [23], who examined the hygrothermal characteristics of a wall constructed from a patented hempcrete mixture with a dry density of 350 kg/m^3^. A computational model was used to assess heat conduction and retention, as well as vapor diffusion, capillary liquid movement, and moisture retention. Unrendered hemp concretes exhibit diverse absorption properties.

Nevertheless, there is a lack of studies that analyze the use of natural fiber in concrete. In this study, the thermal conductivity of concrete with natural fiber is analyzed. Hemp fiber is incorporated into high-density mixtures. Also, samples with synthetic fiber and without fiber are tested to analyze the effect of hemp fiber under the experimental conditions applied.

### Concrete Thermal Conductivity Measurement Methods

Various stationary and transient techniques exist for measuring the thermal conductivity of materials, many of which have historically been applied to rock and soil materials [24,25]. Experiences with rocks and soils [26] indicate that three elements can influence the resulting thermal conductivity values: the chosen method, material composition, and environmental conditions. 

The heat transfer of materials is classified into steady and transient states [27]. A steady state is a constant heat transfer, whereby the temperature or heat flow is not dependent on time. The transient method is dependent on time and temperature changes over time. The methods selected for thermal conductivity measurement differ based on the following two fundamental heat transfer conditions [28]. The steady-state approach is often preferred when dealing with uniform materials. Although this method requires more time, it yields a more precise value for thermal conductivity (k-value) compared to the transient method. On the other hand, the transient method is typically employed for heterogeneous materials that contain moisture [29]. The methods commonly used in the steady state are Boxes and Hot Plane, while for the transient state, the methods used are Hot Wire and Transient Plane Source. In [28], different k-value measurement methods used in 30 studies are summarized, and it was determined that 47% use the Hot Wire method, 30% use the Hot Plate method, 13% use the Transient Plan Source, and 10% use the Hot Box.

**Figure 1:**
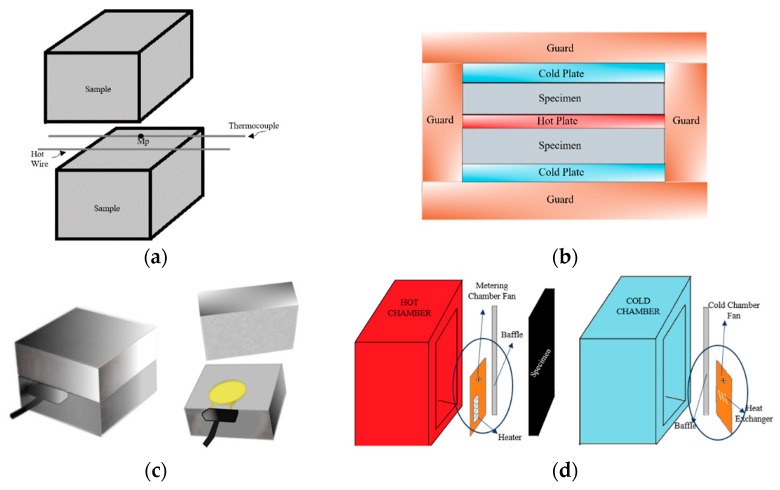
Schemes of method described. (**a**) Hot Wire method. (**b**) Plate Technique. (**c**) Transient Plane Source. (**d**) Hot Box method.

The Hot Wire method is a dynamic approach that involves measuring the elevated temperature at a specific distance from a hot wire, which serves as a linear heat source embedded within the test material (Figure 1a). The hot wire probe method is an application of the transient hot wire technique [30]. Researchers have utilized this method to assess the thermal conductivity (k-value) of concrete using cement replacements such as bottom ash, fly ash, and silica fume, as well as air-permeable concrete and various types of lightweight aggregate concrete [9,14,31,32,33,34,35,36,37,38,39].

The Hot Plate technique is a valuable method for evaluating the thermal conductivity of insulation materials. This method involves placing the test samples between heated and cooled plates (Figure 1b). A constant heat flow is directed over the test samples. The thermal conductivity is determined by analyzing the heat flow and the temperature difference across the surfaces of the specimen [40]. Gandage et al. [29] employed this technique to measure the thermal conductivity of specimens within five temperature ranges, ranging from 30 °C to 80 °C. In another study [41], the temperature of the cooled plate was set at 18 °C while that of the heated side was maintained at 40 °C. Several researchers have employed this technique to determine the thermal conductivity of various types of concrete, including self-consolidating concrete, oil palm shell foamed concrete, oil palm shell foamed geopolymer concrete, aerated lightweight concrete, recycled glass concrete, and polystyrene foamed concrete [29,41,42,43,44,45,46].

In the Transient Plane method, the thermal conductivity is measured based on the power input and time-dependent variation for both transient plane and transient line sources. The transient plane source (TPS) method is used to measure the in-plane and through-plane thermal conductivity of materials. In this method, a flat sensor is used, the temperature of which is increased while an electrical current passes through the sensor (Figure 1c). By recording the temperature against time, it is possible to calculate the thermal properties of the material [28]. The advantage of the TPS technique is the simplicity of the equipment, and simultaneous information on thermal conductivity [47]. Also, transient techniques offer a significant edge over steady-state techniques due to their ability to eliminate the impact of contact resistance when evaluating experimental data. This elimination process enhances the precision of measurements across a diverse spectrum of thermal conductivities and, by extension, a wide array of materials. The added advantage of having TPS sensors shielded by a polymer coating is that it facilitates measurements even on electrically conductive materials. Nonetheless, in the ongoing study, the primary focus is on employing the TPS technique to elucidate the concepts of thermal conductivity and specific heat primarily concerning insulation materials [48]. 

The Hot Box is a method that maintains a consistent condition for determining the thermal conductivity of concrete. It relies on evaluating the energy within the system. This technique was developed in the laboratory for analyzing thermal and solar properties at Claude Bernard University-Lyon I in France. The setup consists of two chambers: one that is heated and another that is cooled (Figure 1d). The concrete sample is positioned between these chambers. Following the principles of the second law of thermodynamics, energy flows from the heated side to the cooled side. By calculating the temperature disparity between the cooled and heated sides, the value of thermal conductivity (k-value) can be determined [49,50].

The utilization of specific methods and devices in research laboratories may depend on the availability of equipment. Moreover, different sample shapes and sizes are employed based on the testing requirements of a specific device. However, the thermal conductivity of concrete is not significantly influenced by the shape and size of the specimen [51].

In this study, the Needle method, or the Transient Line Source method (TLS), was selected. The scientists primarily assessed the thermal properties of materials using the Guarded Hot Plate (GHP) method before developing the TLS method. This technique must be conducted under laboratory conditions, often requiring extended test times and large sample sizes; hence, the concept of a highly portable transient probe that required minimal setup was highly desirable. The robust design of the TLS probe allows for testing both in the laboratory and in the field. Compared to other techniques, the TLS method can test a wide range of materials, including soils, rocks, concrete, polymers, moist and porous materials, and even liquids [52,53]. One of the main advantages of the TLS method is its portability. The rugged design enables this method to provide accurate in situ and laboratory measurement results with a simple sample setup. Compared to other methods, the TLS technique is fast, user friendly, and reliable. This method has proven to be effective even when assessing porous materials containing moisture [52,54]. Steady-state methods cannot account for the variability that moisture evaporation and condensation have on thermal conductivity, and therefore, the transient line method provides the most accurate results.

**Figure 2:**
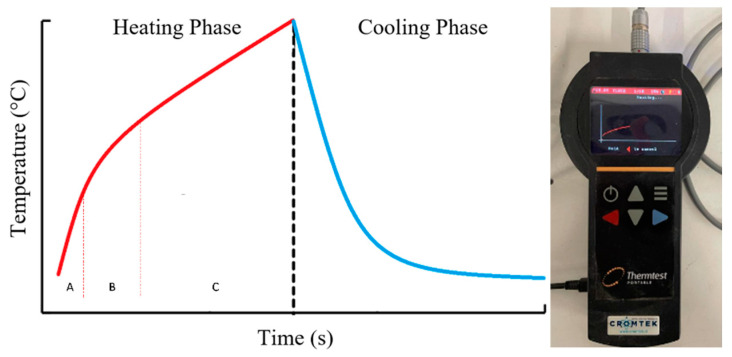
Typical form of the temperature versus time graph for the simple needle test. Heating and cooling phase equipment.

The system consists of a measuring unit (Figure 2—right), a needle-shaped heating element, and a temperature sensor. The procedure with the thermal needle includes two phases. In the first phase, known as the heating stage, the needle is inserted into the soil sample and allowed to rest for a certain period to achieve thermal equilibrium with the surroundings. Subsequently, an electric current of known magnitude is applied, inducing the injection of heat at a constant rate. This process leads to an increase in the ambient temperature, which is recorded at short time intervals. In the second phase, known as the recovery or cooling stage, the heat source is disconnected, and the decrease in temperature over time is recorded. The typical graphical representation of temperature against time is shown in Figure 2—left. The heating and cooling (or recovery) stages can be subdivided into three stages [55]: (A) The first encompasses the initial seconds of the test and corresponds to the period in which the system’s response is influenced by the thermal properties of the needle. (B) The second represents a semi-steady phase in which the temperature variation is linear with respect to the logarithm of time. (C) The final stage of the test is the effect of temperature at the boundaries of the sample begins to change, no longer adhering to one of the fundamental assumptions of the method.

## 2. Materials and Methods

In this study, the conductivity of the concrete with two types of fiber is evaluated after 7 and 28 curing days using the Transient Line Source method. Also, samples without fibers are tested. In this section, the material of the experimental setup is described. 

### 2.1. Methods and Equipment

**Figure 3:**
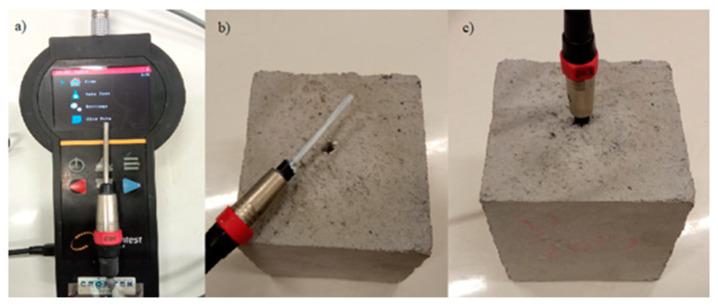
TLS-50 equipment used to measure conductivity and thermal resistance on concrete samples. (**a**) Main equipment device, (**b**) example of concrete sample and sensor needle with thermal paste, (**c**) example of measurement through the hole previously drilled in concrete sample.

The Transient Line Source meter model 50 (TLS-50; Figure 3a) is used here. TLS-50 is a portable meter used to measure the conductivity and thermal resistance of a variety of samples, including soil, rocks, concrete and polymers [56,57]. Also, it is a non-destructive tool used to measure the thermal conductivity of materials such as lightweight concrete. Due to the non-destructive nature of this tool, it is possible to accurately measure the thermal conductivity of concrete and without affecting its structural integrity (Table 1). This equipment can instantly display the results once the samples are ready. The sensor needle consists of a heating wire (thin), and a temperature sensor sealed in a steel tube measuring 150, 100 or 50 mm; the last one is used in this study (Figure 3b). The sensor is fully inserted into the sample to be analyzed (Figure 3c).

**Figure 4:**
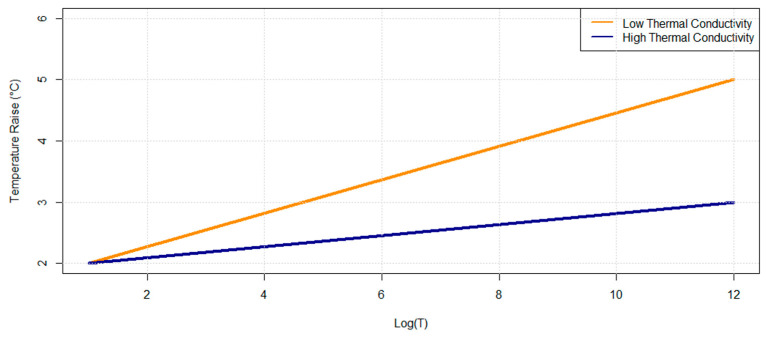
Low and high thermal conductivities.

In this equipment, heat is delivered to the sample by a constant current source (*q*) and the temperature increment is recorded over a period defined. The thermal conductivity (*k*) is calculated using a slope (*a*) between the temperature increment and the logarithm of time (Figure 4) using Equation (1).

(1) $$k=\frac{q}{4\pi a}$$

In Equation (1), *k* is the thermal conductivity (W/m°K), *q* is the heat power, and *a* is the slope of the curve. The higher the thermal conductivity of a sample, the less steep the slope will be [58].

For hard samples, such as rock and concrete, the 50 mm needle test and a 4 mm diameter bit must be used. During tests, heat-dissipating grease is used to optimize the contact between the sensor and the sample. 

### 2.2. Materials

#### 2.2.1. Cement

**Figure 5:**
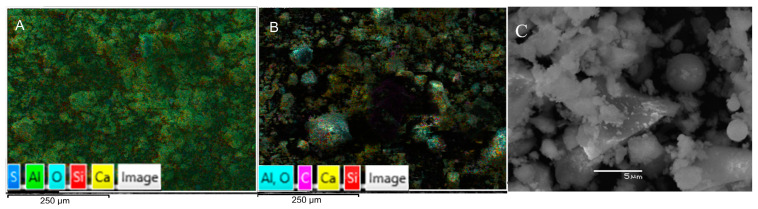
SEM of pozzolanic cement ((**A**,**B**): 250 μm scale (mapping), and (**C**): 5 μm scale).

A pozzolanic class cement was used, according to its composition and strength [59], from Cementos Biobio, Chile. The outcomes of an Energy-dispersive X-ray spectroscopy (EDS) analysis offer insights into the elemental makeup of cement. Generally, EDS findings for cement will reveal the existence of calcium, silicon, aluminum, various oxides, and iron as the primary constituents within the cement. These elements constitute the fundamental building blocks of cement and play a crucial role in conferring the cement’s strength and bonding characteristics. Figure 5 displays the Scanning Electron Microscopy (SEM) morphologies of the cement on scales of 250 and 5 um, where elements such as calcium and aluminum appear.

**Figure 6:**
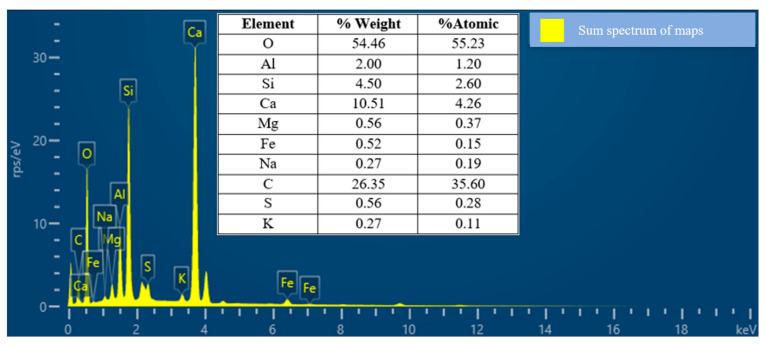
EDS of pozzolanic cement.

Figure 6 shows, through the EDS images, the weight concentration of elements in the pozzolanic cement. The SEM/EDS results mainly specify the chemical composition of the pozzolanic cement materials present. The elemental composition of pure oxygen (O) was the most abundant element, accounting for 54.46% of the total weight, followed by carbon (C), at 26.35%, and calcium (Ca), which accounted for 10.51%, and the composite pozzolanic cement was estimated by the EDS technique. Depending on the location of the high-concentration contents’ dispersion, the chemical and mechanical properties of the mortar will harden, evidently showing a good C-S-H form in the mixture, which directly influences the stronger pozzolanic activity during the hydration process.

**Figure 7:**
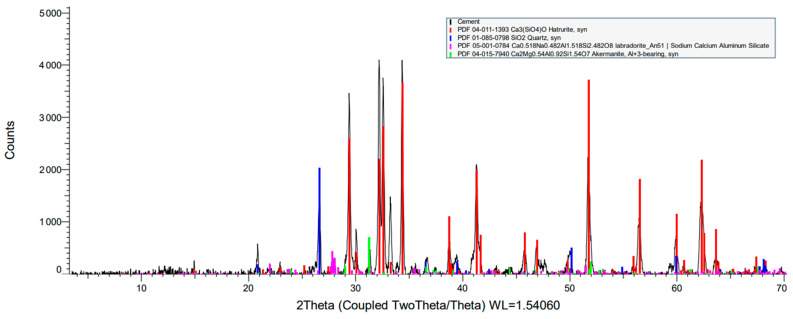
X-ray diffraction (XRD) of pozzolanic cement.

In Table 2, there can be seen four types of composition components, which are hatrurite, quartz, ferrite, labradorite and akermanite. In Figure 7, the overlapping of the diffraction peaks of the main component, Hatrurite, in the 2θ = 3° to 70° range, can also be seen.

According to Figure 7, which shows the obtained X-Ray diffraction (XRD) graph, the most prevalent components in pozzolanic cement specimens were silicon, aluminum, iron, oxygen, and calcium, etc. Due to their possible pozzolanic and semi-cementitious properties, silica and alumina have a positive impact on good cement alternatives. The images in Figure 6 show the particle distribution for pozzolanic cement specimens at a size of 100 nm. According to [60], the content of the three oxides combined, Fe_2_O_3_, Al_2_O_3_, and SiO_2_, should be greater than 70% for natural pozzolanic cement to be suitable for employment in the blend. XRD studies have verified that sustainable concrete can withstand the thermal conductivity according to the chemical examinations. This conclusion implies that the primary morphological patterns are rather widespread in pozzolanic cement.

#### 2.2.2. Aggregates

**Figure 8:**
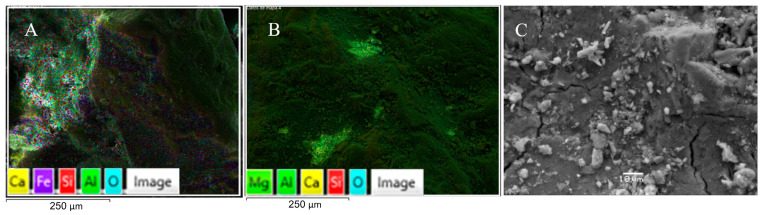
SEM of sand ((**A**,**B**): 250 μm scale (mapping), and (**C**): 10 μm scale).

The sand used is ACBB coarse sand, which was subjected to pycnometer test, and sieving test. The pycnometer procedure is performed twice from 10 g of sand, obtaining an average value for density equal to 2.76 g/cm^3^. Figure 8 shows the SEM analysis, which shows elements like calcium, carbon and aluminum.

**Figure 9:**
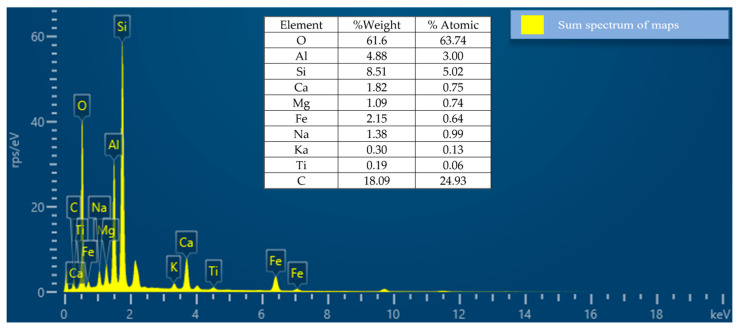
EDS of sand.

Figure 9, which presents EDS images, shows the weight concentration of elements in the sand. The SEM/EDS results mainly specify the chemical composition of the sand materials present. The elemental composition of pure oxygen (O) was the most abundant element, accounting for 61.6% of the total weight, followed by carbon (C) at 18.09% and silicon (Si), which accounted for 8.51%. There was a high presence of elements such as oxygen, silicon, and carbon in addition to the organic content of the sample. 

**Figure 10:**
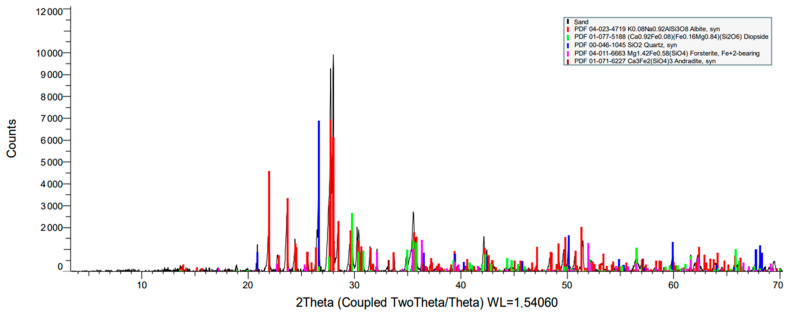
X-ray diffraction (XRD) of sand.

Table 3 and Figure 10 show the XRD analysis, indicating the most prevalent components in natural aggregate specimens. Due to their possible silica and cementitious properties, these components have a positive impact. The particle distribution for natural aggregate is 100 nm in size, according to [61], and Fe_2_O_3_, Al_2_O_3_, and SiO_2_ are suitable for employment in the blend in sustainable concrete with the aim of withstanding the thermal conductivity. This conclusion implies that the primary morphological patterns are rather widespread in natural aggregate.

**Figure 11:**
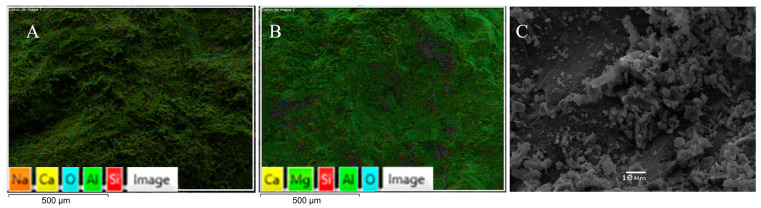
SEM of coarse aggregate ((**A**,**B**): 250 μm scale (mapping), and (**C**): 10 μm scale).

In the case of gravel, sizes between 10 mm and 4.75 mm were used, using approximately 5% of the total amount in the transition from sand to gravel. Figure 11 shows the SEM results, which show elements like sodium, calcium, and aluminum. 

**Figure 12:**
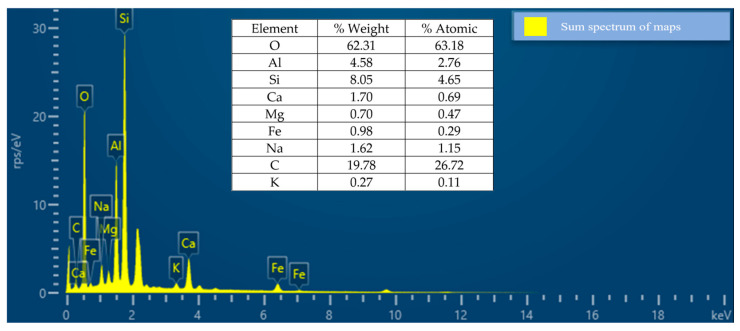
EDS of coarse aggregate.

Figure 12 shows the EDS images, which indicate the weight concentration of elements in the coarse aggregate. The SEM/EDS results mainly specify the chemical composition of the coarse aggregate materials present. The elemental composition of pure oxygen (O) was the most abundant element, accounting for 62.31% of the total weight, followed by carbon (C) at 19.78% and silicon (Si), which accounted for 8.05%.

**Figure 13:**
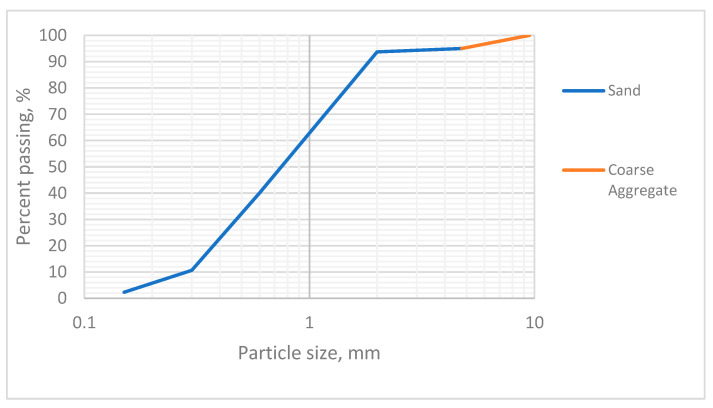
Particle size distribution of the aggregate used.

For this case, gravel from a local quarry was used, the size of which ranges from 5 mm to 20 mm. Additionally, most of the gravel was crushed in a jaw crusher to obtain a defined particle size distribution (Figure 13). Finally, both aggregates comply with the standard [62].

#### 2.2.3. Fibers

Two fibers were used in this study: a synthetic fiber BarChip 54 model, which is commonly used for shotcrete in underground mining (BarChip54); and a natural fiber of hemp. The synthetic fibers are certified by the European CE standard under the requirements of performance of the EN 14889 standard [63] and by the ASTM C1116 standard [64] type III [60]. 

**Figure 14:**
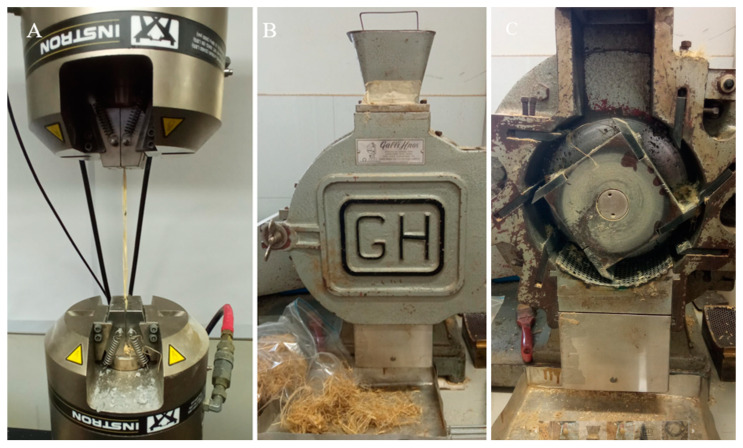
(**A**): Hemp fiber tensile strength measurement, (**B**,**C**): blade mill used for cutting fibers.

Table 4 shows some relevant parameters of the fibers utilized in this study. The length and diameter of natural fiber is very variable after milling. The natural fiber length and diameter were measured using a ZEISS Stemi 2000-C microscope and AxioVision REL 4.8. software. The diameter of the fiber used here is slightly larger than that of others reported [65]. Also, the tensile strength of the hemp fiber was measured (Figure 14A) to compare with that of the synthetic fiber. Then, the fiber was cut using a blade mill (Figure 14B,C).

**Figure 15:**
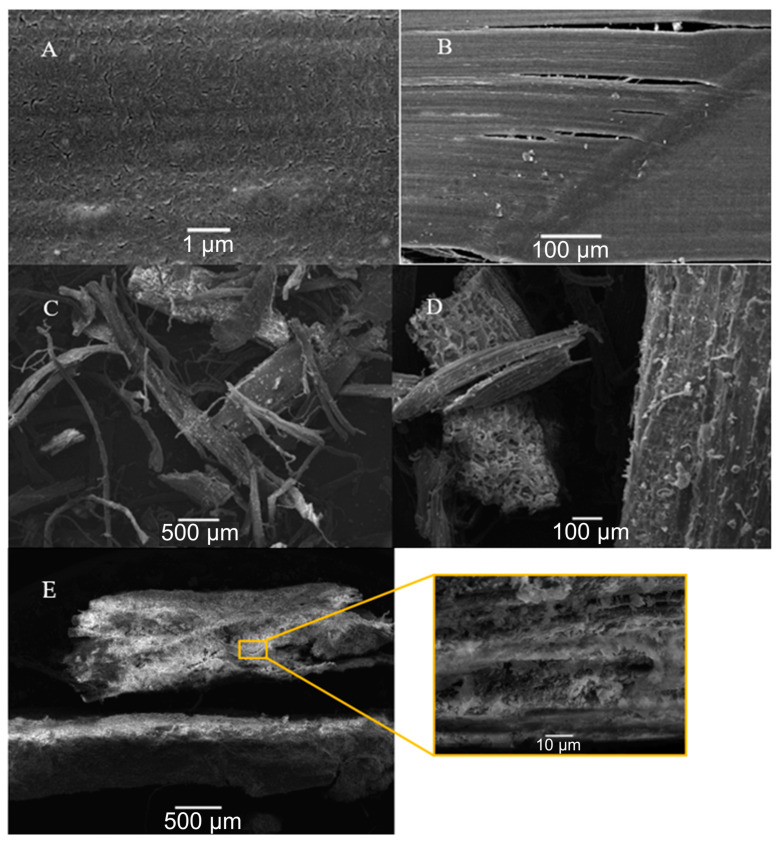
SEM of fibers (synthetic, (**A**): 100 μm scale, (**B**): 250 μm scale; hemp, (**C**): 100 μm scale, (**D**): 500 μm scale, (**E**): 500 μm and 10 μm scale).

**Figure 16:**
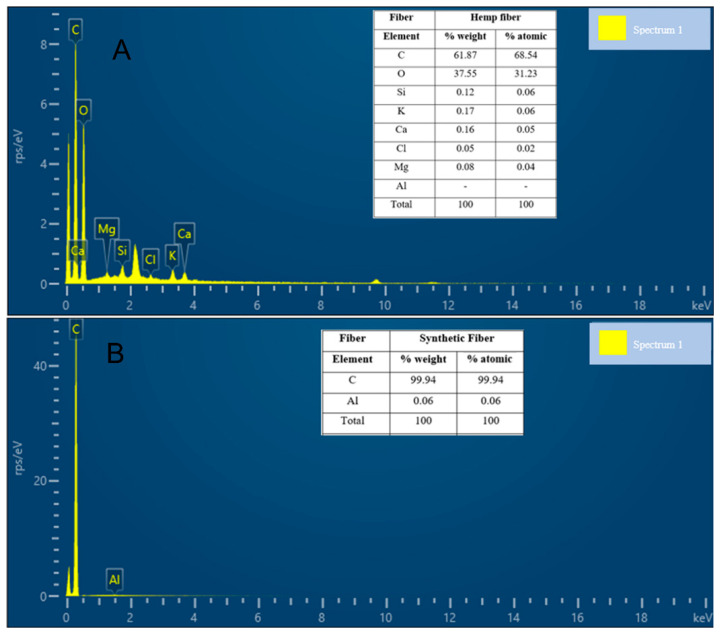
EDS results of hemp (**A**) and synthetic (**B**) fibers.

Additionally, SEM and EDS tests were used to analyze the fibers’ morphology, topographic surface, and composition. Figure 15 shows the SEM images taken at different scales of the fibers and Figure 16 shows the weight concentration of elements in the fibers. 

The SEM/EDS results mainly specify the chemical composition of the fiber materials present. The elemental composition of carbon (C) was the most abundant, accounting for 61.87% of the total weight of hemp and 99.94% of that of the synthetic fiber.

### 2.3. Sample Preparation and Testing Procedures 

Eighteen concrete mixtures were prepared with a dosage of 1.2% fiber to the weight of cement and with a water–cement ratio of 0.5 to analyze the thermal conductivity according to [56]. This percentage was selected because it has shown good results in the mechanical behavior of concrete. The thermal conductivity of the nine mixtures was evaluated using cube samples of 105 mm in length (Figure 3b,c). Curing took 7 and 28 days in water at a temperature of 20 ± 1 °C. Then, the cubes samples were dried in an oven for 24 h at 110 ± 5 °C. Then, they were left to cool at 21 ± 2 °C. After preparing the concrete mixtures and leaving these in the molds, a nail with a 4 mm diameter was inserted to create a hole in the center of the samples. The test needle must be covered with a thermal paste to improve sensitivity and have better thermal contact between the wave sensor and the dry concrete sample before using TLS-50. Details of the samples are shown in Table 5, as well as the results obtained. For the patron repeats, the samples were named P1, P2 and P3. The same process was carried out for the samples with hemp fiber (H) and those with synthetic fiber (S) for each curing day.

During the tests, a test time of 60 s and a heat current of 250 mA were selected. Also, a waiting time of 1 min per measurement was utilized. The number of tests per measurement was 8:4 measurements for the concrete sample and 4 measurements for the equipment pattern (8 measurements per concrete sample). 

## 3. Results

**Figure 17:**
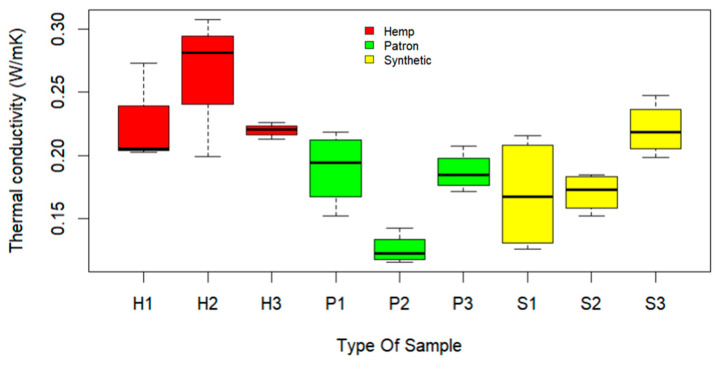
Conductivities with current of 250 mA at 7 days.

**Figure 18:**
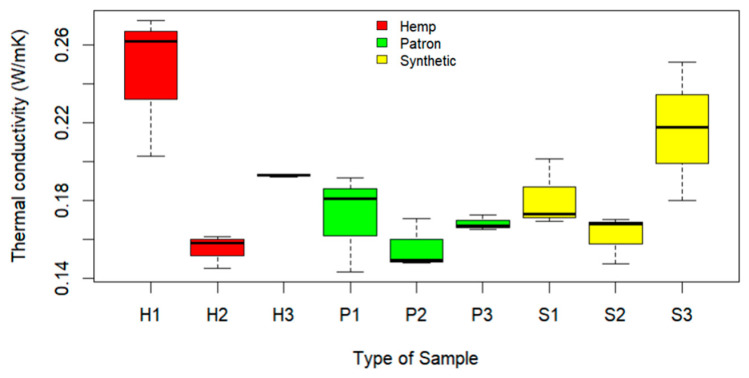
Conductivities with current of 250 mA at 28 days.

Figure 17 and Figure 18 show the thermal conductivity results obtained by the TLS-50 equipment when using a current of 250 mA and a measurement time of 60 s on concrete samples with 7 and 28 days of curing. In the graphs, the red color represents hemp (H), the green represents the reference sample (P), and the yellow represents the synthetic fiber (S). 

Figure 17 shows the results for 7 days of curing. Here, hemp fibers exhibit the highest thermal conductivity values, especially in the case of hemp sample 2 (H2), and hemp sample 3 (H3) showed more accurate results. The reference samples (P) display lower thermal conductivity values compared to the hemp samples. The samples with synthetic fibers show significant similarities with the reference samples in terms of thermal conductivity, except for synthetic fiber sample 3 (S3), which bears similarities with the values of the hemp fiber samples.

The measurements of thermal conductivity were carried out on samples cured for 28 days (Figure 18) using the same parameters as in the previous case. In the first hemp sample (H1), the highest thermal conductivity value was recorded. However, this behavior was not observed in the other two hemp fiber samples, with hemp sample 2 registering one of the lower values. This behavior could be related to the fiber location in the samples. The reference samples (P) show lower results compared with hemp fiber samples H1 and H3. The reference samples showed similar values between them. The synthetic fiber samples (S), as in the 7-day curing period, exhibited a behavior similar to that of the reference samples.

## 4. Discussion

**Figure 19:**
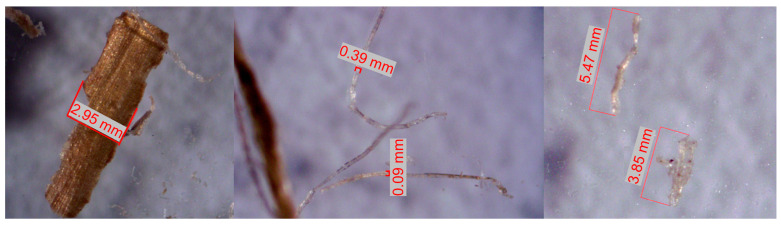
Hemp samples measured with the ZEISS STEMI 2000-C microscope along with AxioVision software REL. 4.8.

The thermal conductivity generally decreases from 7 to 28 days of curing. This may be attributed to the quality of the hemp fiber. The hemp fiber was ground using a blade mill (Figure 14B,C), resulting in different thicknesses, densities, and fiber lengths (Figure 19). Consequently, a lower fiber density led to a lower thermal conductivity in the sample, explaining the variability in the results.

Furthermore, the reduction in thermal conductivity instead of an improvement with the addition of hemp fibers can be attributed to the air trapped within the hemp fiber during mixing, as air pockets can create numerous weak cavities in smaller fibers [66]. The decrease in thermal conductivity at 28 days of curing is due to the extended presence of hemp fiber in the sample at that dosage. Hemp may not always benefit the concrete sample by reducing its conductivity as it becomes vulnerable to bacterial attacks [65], a situation that may have occurred for sample H2, specifically, at 28 days of curing. Additionally, there is a possibility that the water absorbed by the fibers, in significant quantities, remains available and is utilized for further cement hydration [67], leading to the results at 7 days of curing. In addition, in the SEM micrographs (Figure 15C,D), it was observed that hemp fibers exhibit a triangular-like geometric shape, and are composed of cellulose, hemicellulose, and lignin, similar to what was presented in [68]. Figure 15E reveals that the surface of cellulose microfibrils exhibits good adhesion with cement due to its superficial texture, and they are uniformly distributed in the cement concrete. Additionally, a stronger bond between hemp fiber and the concrete matrix was noted. While good adhesion was achieved in samples H2 and H3, the thermal conductivity did not experience significant improvements because hemp fiber tends to trap air within the matrix, creating pores.

In the patron samples, adjusting the amount of gravel to 5% of the coarse sand, a thermal conductivity result similar to that of lightweight concrete was obtained. The typical range of thermal conductivity for lightweight concrete is 0.2 to 1.9 W/m°K, whereas for conventional concrete, it is 0.6 to 3.3 W/m°K [11,69,70,71,72]. Therefore, the reference sample shows low thermal results, close to those of lightweight concrete due to the percentages of the aggregates used.

In a study by Fraternali et al. [73], which investigated recycled polyethylene terephthalate fibers and virgin polypropylene at a 1% dosage in concrete, they found that the thermal conductivities were reduced compared to those of normal concrete. It is not only synthetic fibers that affect the thermal conductivity of fiber-reinforced concrete, as small permeable voids [41,74] can also restrict the heat transfer. Furthermore, as more volume fractions of synthetic fibers are mixed into concrete, more permeable voids are found in fiber-reinforced concrete [75,76]. Another study [77] shows that plastic fibers, including polyethylene fibers, can decrease the thermal-inducing property or heat transfer of produced concrete.

It can be observed that the addition of polypropylene synthetic fiber does not reduce the thermal conductivity of the concrete sample, contrary to what is mentioned by the cited authors. This fiber has a length of 50 mm, and perhaps this length and the distribution of the fiber in the matrix prevent the formation of pores in the sample to such an extent that better thermal conductivities can be achieved, as shown by sample S3 for both 7 and 28 days of curing.

Finally, Table 6 shows different types of concrete samples, with different water–cement ratios, densities, and thermal conductivities. The samples in this study have densities close to that of conventional concrete, but the values of the thermal conductivities are close to that of lightweight concrete. Thus, the volume fraction is a key factor in determining the thermal conductivity of concrete. It should be noted that this study did not consider the percentage of cellulose and lignin when using hemp natural fiber, and thus, this is not reflected in Table 6. However, the chemical composition of these elements plays a significant role in the concrete matrix, influencing thermal conductivity.

## 5. Conclusions

This study explores the behavior of thermal conductivity in concrete containing two different types of fiber. In particular, the concrete samples were analyzed using the Transient Line Source method, which is a time-efficient technique for testing this type of material after a calibration process. During the manufacturing process of the mixtures, a homogenization step was conducted to ensure a uniform distribution of the fibers within the matrix. They were then cured for 7 and 28 days prior to the evaluation of their thermal conductivity. The results were compared to those of concrete without fibers to quantify the impact of the fibers on the thermal conductivity. The results revealed that the incorporation of hemp fiber in the concrete resulted in a 46% increase in thermal conductivity after 7 days of curing, as shown by samples H2 and P1, and in a 43% increase after 28 days of curing, as shown by samples H1 and P1. On the other hand, the concrete with synthetic fiber did not show major differences compared to samples without fiber. It is considered that although hemp fiber may introduce air into the concrete matrix, it exhibits good adhesion to the concrete, increasing water absorption and resulting in higher thermal conductivity compared to the rest of the samples. These findings provide additional evidence offering a natural alternative, such as hemp fiber, to increase the thermal properties of concrete and thus adapt concrete constructions according to the required thermo-active foundations and geothermal energy piles.

## Figures and Tables

**Table 1:** Main TLS-50 equipment parameters and description [58].

Materials	Concrete, Rocks, and Polymers
Measuring Capacity	Bulk Properties
Thermal Conductivity	0.3 to 5 W/m°K
Thermal Resistivity	0.2 to 3.3 m°K/W
Measuring Time	1 to 90 Minutes
Reproducibility	±2%
Precision	±5%
Temperature Range	−40 to 100 °C
Minimum Sample Size	large 50 mm, diameter 50 mm

**Table 2:** Results of automatic semi-quantification (S-Q) of cement.

	Semi-Quantitative Mineralogy			
**Sample**	Plagioclase	Quartz	Hatrurite	Akermanite
**Cement (%)**	10.2	14.5	68.7	6.6

**Table 3:** Results of automatic semi-quantification (S-Q) of sand.

	Semi-Quantitative Mineralogy				
**Sample**	Plagioclase	Pyroxene	Quartz	Olivine	Garnet
**Cement (%)**	64.9	13.1	11.8	8.5	1.7

**Table 4:** Physical characteristics of fibers used.

Parameter	Synthetic	Hemp
Tensile strength, MPa	640	0.052 ± 0.0046
Fiber length, mm	54	2.45 ± 1.25
Fiber diameter, mm	0.70	0.1 ± 0.06

**Table 5:** Percentage of materials used.

	Materials (%)				7-Day Density (g/cm^3^)		28-Day Density (g/cm^3^)	
**Sample**	**Sand**	**Cement**	**Coarse** **Aggregate**	**Fiber**	**Wet**	**Dry**	**Wet**	**Dry**
**Patron (P)**	64.55	32.27	3.18	0.00	2.43	2.26	2.37	2.25
**Synthetic (S)**	64.30	32.15	3.16	0.39	2.34	2.18	2.31	2.18
**Hemp (H)**	64.30	32.15	3.16	0.39	2.41	2.23	2.38	2.25

**Table 6:** Summary of different studies of thermal conductivity in concrete samples.

Type Sample	*w*/*c*	Dry Density (kg/m^3^)	(W/m°K)	References
Concrete with recycled coarse aggregate	0.35	2112	0.98–0.99	[78]
	0.45	2087	0.88–0.89	
	0.5	2075	0.76–0.77	
	0.55	2069	0.69–0.70	
Concrete with rock aggregate	-	-	1.163–8.6	[9]
Lightweight concrete	-	-	0.2–1.9	[11,70,71,72]
Normal weight concrete	-	-	0.6–3.3	
Structural lightweight aggregate concrete	-	1850	0.58–0.86	[72]
	-	1400–1800	0.85–1.05	
Cement mortar with 10%, 20%, 50% of recycled high-impact polystyrene as a sand substitute	-	-	0.53	[79]
	-	-	0.42	
	-	-	0.27	
Dune sand concrete	-	2100	1.2	[80]
Dune sand concrete with wood shavings aggregate	-	1400	0.55	
Lightweight concrete made of tobacco waste	-	-	0.19–0.22	[81]
Reference concrete	0.5	2240	2.24	[82]
Magnetite concrete	-	3650	2.57	
Graphite concrete	0.59	1890	3.52	
Graphite and magnetite concrete	0.6	2810	3.85	
Steel fiber concrete	-	2330	2.57	
Steel fiber concrete with high concentration of fibers	-	2441	2.95	
Concrete with brass shavings	-	2520	2.71	
Concrete with copper wires	-	2438	3.63	
Concrete with PCM pellets	0.5	1790	1.23	
Concrete with micro PCM	-	1570	0.97	
Concrete with PCM dispersion	-	1900	1.31	
Sprayed hemp concrete	-	374–450	0.116–0.145	[22]
Reference (patron) concrete (28 curing days)	0.5	2250	0.143–0.192	This study
Concrete with hemp fibers (28 curing days)		2250	0.145–0.273	
Concrete with synthetic fibers (28 curing days)		2180	0.148- 0.251	

## Data Availability

Data are contained within the article.
